# Illinois General Hospital

**Published:** 1851-03

**Authors:** 


### ARTICLE II.

### ILLINOIS GERERAL HCSPITAL.

This institution has now been open for the reception of patients since November. The project of opening it in the Tippicanoe Hall, was abandoned and the Lake House was secured in its stead.

This establishment known throughout the North West as one of the largest and finest tavern edifices in the western country, will afford ample room and admirable conveniences for the care of 200 patients.

An arrangement has been made by which the "Sisters of Mercy" have the care of the Institution, who took charge of it in February ult. The admirable neatness and air of comfort that are everywhere manifest in the Institution prove the wisdom of their appointment.

The Physicians and Surgeons that have been appointed are D. Brainard, M. D., Surgeon; N. S. Davis, M. D., and Levi D. Boone, M. D., Physicians; John Evans, M. D., Physician to treat diseases of females.

Other appointments it is understood will be made in a few days.

The institution has the care of the sick paupers of the country who are suitable objects of hospital treatment, and also of the marine patients, until the Marine Hospital shall be opened for their reception.

We would advise our readers in Illinois that by the law establishing the Institution, (which may be found in the acts of the extra session of 1849) the county boards of each county in the State, are authorized to send patients to the Institution at county charge, which will enable them to put under the treatment of the Institution any cases that may be thought advisable. The price of admission to such patients, including board, medical treatment, &c., has been fixed at $2,50 per week.

Private patients can have separate apartments, and will be received at prices varying according to the accommodations at from $4,00 per week.

Our friends may rest assured that patients whom they may send to the Hospital will receive every necessary attention and comfort.

Our readers, too, may expect regular reports of clinical lectures, and interesting cases in the Journal.

Students are admitted to witness practice in the wards.

